# Evaluation of mandibular growth in a prepubertal Class I and Class II population: a longitudinal analysis

**DOI:** 10.1093/ejo/cjae014

**Published:** 2024-03-20

**Authors:** Lucia Pozzan, Denis Guerra, Giulia Ulian, Giorgia Checchin, Roberto Di Lenarda, Luca Contardo

**Affiliations:** Department of Medicine, Surgery and Health Sciences, University of Trieste, Trieste, Italy; Private practice, Vicenza, Italy; Private practice, Trieste, Italy; Department of Medicine, Surgery and Health Sciences, University of Trieste, Trieste, Italy; Department of Medicine, Surgery and Health Sciences, University of Trieste, Trieste, Italy; Department of Medicine, Surgery and Health Sciences, University of Trieste, Trieste, Italy

**Keywords:** Mandibular growth, prepubertal growth spurt, cephalometric analysis

## Abstract

**Objective:**

This study aims to identify the presence, timing, and magnitude of a prepubertal mandibular growth spurt in a Class I and Class II population.

**Methods:**

From the Burlington and Iowa Growth study of the AAOF Craniofacial Growth Legacy Collection, 83 Class I subjects (37 females and 46 males) and 32 Class II subjects (18 males and 14 females) were identified, as having at least seven consecutive annual lateral cephalograms taken from 5 to 11 years of age. Only subjects with a normodivergent facial pattern were considered. A customized cephalometric analysis was performed, and total mandibular length, defined as the distance between Condylion (Co) and Gnathion (Gn), was calculated.

**Results:**

Overall, a significant early peak of mandibular growth was present in all the subjects analysed both in Class I (4.69 mm for males and 4.18 mm for females; *P* < .05) and in Class II (5.85 mm for males and 4.05 mm for females; *P* < .05). No differences between males and females were found for the timing of this peak (7 years for Class I and Class II females and 7 years for Class I and 6.5 years for Class II males). In males, a significantly larger peak was observed in Class II than Class I subjects (*P* = .007).

**Limitations:**

The main limitations of this study were the impossibility of using a suitable growth indicator to identify the timing of the early mandibular growth peak and the limited Class II records retrievable.

**Conclusion:**

This investigation suggests that a prepubertal mandibular growth peak is consistently present in both Class I and Class II males and females of clinically significant magnitude. Despite that, chronological age confirms to be unsuitable to identify this peak.

## Introduction

The timing of facial growth and the identification of stages of skeletal maturation have been the subject of many investigations. This interest comes from a large body of evidence that shows how the greatest effect of functional therapy in skeletal Class II malocclusions due to mandibular retrusion is achieved when the peak in mandibular growth is included in the treatment period and that more favourable orthopaedic outcomes can be expected when the treatment is undergone after the start of the growth spurt [1–7]. When starting treatment in the optimal maturation stage, that is, the circumpubertal period, it is possible to optimize the results while limiting undesired effects [8–10].

Many cephalometric studies have analysed the peak in mandibular growth during the pubertal period (pubertal growth spurt) [11–13], and have investigated its correlation with growth indicators, in particular standing height, the Hand-and-Wrist Method (HWM) [14], the Cervical-Vertebral Method (CVM) [8], and the Middle Phalanx maturation Method (MPM) [15]. Some investigations underlined how, for most of the patients analysed, the adolescent peak in mandibular size occurred simultaneously or slightly after the peak in standing height [11, 16, 17]. Other studies showed that this growth peak follows its onset by about 2 years [16, 18–21].

All the mentioned investigations have one main aspect in common: the observation of a great interindividual variation regarding the intensity, onset, and duration of this growth peak [11, 12]. One example is the observation that early-maturing children, considered those with advanced skeletal age, have a corresponding early circumpubertal growth spurt, while late-maturing children have a delayed growth spurt [18, 22].

Even if the largest part of the literature has focussed on the study of the pubertal growth spurt, growth accelerations for various somatic quantities were also identified at younger ages [11, 23–26]. Gasser defined such periods of early growth acceleration as mid-growth spurt (MS) and placed it at approximately 7.5 years in both sexes. These mid-growth spurts were identified in most patients, about two-thirds [27, 28]. A more recent investigation reports an evident prepubertal spurt for girls, that closely precedes the pubertal growth spurt, while no clear conclusion could be gathered for boys, where a relatively constant growth rate seems to stand out up to the onset of the spurt [21].

Since the goal of Mellion’s study was to collect a large longitudinal sample to develop incremental growth curves for several parameters, no distinction was done based on the facial growth pattern or type of malocclusion of the subjects [21]. As Montasser previously pointed out in 2019 [29], this broad inclusion can be considered a weakness of this investigation and of previous studies [8, 30, 31]. Mandibular growth in Class II Division 1 malocclusion is smaller compared to Class I subjects [31], while Class III malocclusion shows greater mandibular growth and longer peaks than those in Class I [6, 32–34].

Therefore, this study aimed to perform a longitudinal analysis on cephalometric records of Class I and Class II subjects from 5 to 11 years of age to identify the presence, entity, and timing of an early mandibular growth spurt. For the assessment of mandibular length, we chose the Condylion (Co)- Ganthion (Gn) dimension, since previous investigations discouraged the use of other landmarks, such as Ar-Gn or Ar-Pg, because the Articulare point is not considered an anatomic landmark [35–37].

The null hypothesis is that in both samples there is no statistically significant difference in any of the annual increments between 5 and 11 years of age in males and females.

## Materials and methods

Subjects were selected from the the Iowa Growth Study and Burlington Growth Study files, from the American Association of Orthodontists Foundation (AAOF) Craniofacial Growth Legacy Collection (www.aaoflegacycollection.org). Subjects included had a series of consecutive annual lateral cephalograms from 5 to 11 years of age. This age interval was selected based on previous evidence reporting a mean age for the pubertal growth peak between 11 and 13 years for females and between 13 and 14 years for males [38]. The radiographic records had to be of good quality and the cephalometric points clearly recognizable. Subjects with Class I and II skeletal relationships were identified as those having an ANB angle of 1–4 and ≥ 5 [37, 39, 40] respectively, in at least three out of the four first recordings. Additionally, normodivergent subjects were selected, considering a total divergence (as SN-CoGn angle) between 25 and 42°C, as per previous investigations [38]. Exclusion criteria were (i) incomplete records; (ii) radiographic records of poor diagnostic quality; (iii) subjects with visible signs of craniofacial syndromes; and (iv) evident orthodontic treatment, even minimal, such as a space maintainer. From the original samples available in the AAOF Craniofacial Legacy Collection, 83 Class I cases (37 females and 46 males) and 32 Class II subjects (18 males and 14 females) were included. A total of 21 and 62 Class I cases and 22 and 10 Class II subjects were derived from the Burlington and Iowa Growth study collections, respectively. According to the guidelines of the AAOF Legacy Collection, a magnification factor of 10% was adopted for the Burlington Collection files and a variable magnification between 6% and 12.25% was used for the Iowa Collection, depending on the time the file was produced.

**Figure 1.**
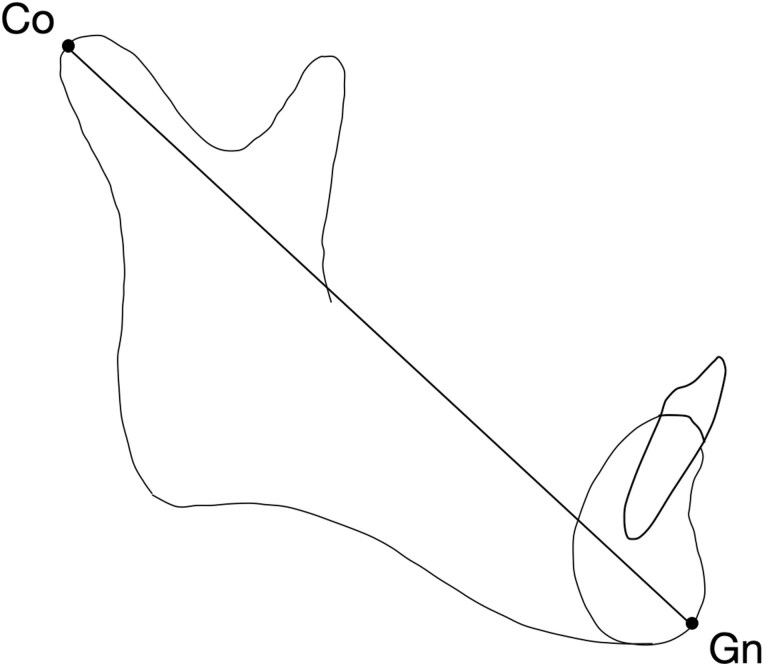
Assessment of mandibular length. Identified landmarks: Co, Condylion and Gn, Gnathion.

A customized cephalometric analysis was performed with Viewbox software (Viewbox version 3.0, dHAL Software, Kifissia, Greece). The analysis was performed by a trained operator, and the accuracy was verified by an experienced orthodontist. Total mandibular length was defined as the distance between Condylion (Co) and Gnathion (Gn) and was calculated for each subject for each radiographic record from 5-year to 11-year record (Fig. 1). Then, annualized increments were derived. Finally, the annual age interval with the greatest increment in Co-Gn distance of the whole series, i.e. mandibular growth peak, was identified.

After the identification of the mandibular growth peak for each subject, data were aligned on this value, and the increments for the 3 years preceding and succeeding the peak were evaluated, as already performed by Perinetti *et al*. [15]. The evaluation of skeletal maturation was not performed, as patients in this age range possess almost exclusively a Cervical Vertebral Maturation (CVM) stages 1 and 2, and a previous investigation showed that a relevant percentage of subjects in the 12–15-year interval experienced a pubertal growth spurt after stages CS1 and CS2 [38].

### Statistical analysis

The method error for the Co-Gn distance was assessed by the method of moments variance estimator and Interclass Correlation Coefficient (ICC) on 30 recordings (15 per collection) randomly selected and expressed as mean (95% confidence interval). Additional analysis on the methos error for the Co-Gn increments was performed on 25 patients randomly selected and is available as supplementary material.

Descriptive statistics were used to characterize the sample. Continuous variables are given as median and interquartile range (IQR). Data were acquired and analysed with SPSS Software (version 26.0, SPSS Inc., Chicago, IL, USA). The failure of the normality assumptions required was verified with the Shapiro-Wilk test. Differences between sexes and between Class I and Class II were tested with the Mann–Whitney *U* test, while the Friedman test and post hoc analysis with Bonferroni correction were carried out to assess the significance of mandibular growth increments at peak with respect to the three preceding and subsequent years. *P*-value was set at .05.

## Results

The method error for the Co-Gn distance was 0.2 mm (0.1–0.6), and the ICC was 0.996 (0.991–0.998), indicating excellent reliability. Results for the method error for the Co-Gn increments are summarized in Table 1.

**Table 1.** Intraclass Correlation Coefficient (ICC) for the Co-Gn increments from 5 to 11 years.

5–6 years	6–7 years	7–8 years	8–9 years	9–10 years	10–11 years
0.85 (0.66–0.94)	0.95 (0.89–0.98)	0.97 (0.92–0.98)	0.96 (0.90–0.98)	0.94 (0.86–0.97)	0.91 (0.80–0.96)

Values are presented as mean and 95% confidence interval (in brackets).

Table 2 shows the distribution of the increments for each yearly intervals for Class I and Class II subjects, divided by gender. For Class I males, median values ranged between 1.32 mm (9–10 years) and 2.91 mm (6–7 years), while for Class II males, values ranged between 1.26 mm (9–10 years) and 3.69 mm (7–8 years). For Class I females, values ranged between 1.17 mm (7–8 years) and 2.73 mm (6–7 years), while for Class II females, the values ranged between 0.89 mm (8–9 years) and 3.33 mm (7–8 years).

**Table 2.** Descriptive statistics for Co-Gn increments for each age interval for Class I and II, divided by sex.

		5–6 years	6–7 years	7–8 years	8–9 years	9–10 years	10–11 years
M	Class I	2.21 (2.68)	2.91 (2.88)	1.74 (2.66)	1.84 (2.87)	1.32 (2.33)	1.37 (2.87)
	Class II	3.48 (3.18)	2.43 (3.69)	3.69 (4.47)	1.8 (1.66)	1.26 (1.88)	1.35 (3.13)
F	Class I	1.59 (2.07)	2.73 (2.44)	1.17 (2.03)	1.44 (2.55)	1.79 (2.58)	1.22 (2.29)
	Class II	1.8 (3.08)	2.16 (2.11)	3.33 (2.57)	0.89 (1.84)	1.69 (1.77)	1.29 (2.23)

F, female; M, male.

Values represent median and interquartile range in brackets.

**Figure 2.**
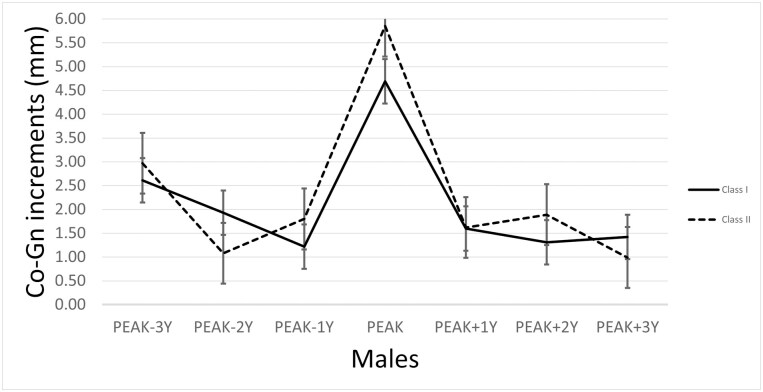
The mandibular growth peak for the male sample, defined as the greatest annualized increment in Co-Go distance, by pooling the Burlington and the Iowa Growth Studies cases and according to the skeletal malocclusion. Data are presented as median ± standard error.

**Figure 3.**
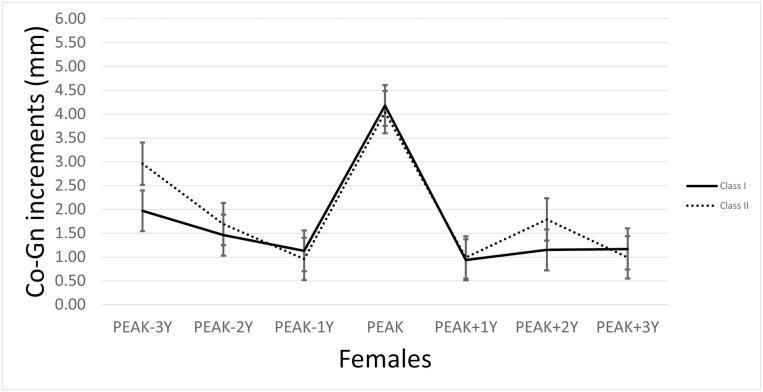
The mandibular growth peak for the female sample, defined as the greatest annualized increment in Co-Go distance, by pooling the Burlington and the Iowa Growth Studies cases and according to the skeletal malocclusion. Data are presented as median ± standard error.

The annualized increments of mandibular growth, plotted aligning the maximum increments (called ‘peak’) [15, 41], divided by sex, are represented in Figs 2 and 3.

For Class I, the values of the highest annualized increments in Co-Gn values ranged from 2.16 mm (Burlington 163, female, 5–6 years of age) to 9.21 mm (Iowa 53, male, 5–6 years of age). For Class II, the values of the highest annualized increments in Co-Gn values ranged from 2.52 mm (Burlington 717, female, 6–7 years of age) to 9.63 mm (Burlington 266, male, 5–6 years of age).

For Class I, the median mandibular growth values at the peak (as median and IQR) were 4.69 mm (1.71) and 4.18 mm (2.35) in males and females, respectively. No significant differences were found between the sexes. All the other median values for the three years preceding and succeeding the peak were generally around 2 mm, ranging from 0.94 mm to 1.97 mm for females and from 1.22 mm to 2.62 mm for males.

For Class II, the median mandibular growth values at the peak (as median and IQR) were 4.05 mm (2.33) and 5.85 mm (2.85) for females and males, respectively. No significant differences were found between the sexes, except for the peak of growth (*P* = .018). All the other median values for the 3 years preceding and succeeding the peak were again around 2 mm, ranging from 0.96 mm to 2.96 mm for females and from 0.99 mm to 2.97 mm for males. A detailed description of the increments at 3 years before and after the peak is reported in Table 3.

**Table 3.** Descriptive statistics for Co-Gn increments for the 3 years before and after the peak.

		PEAK-3Y^†^		PEAK-2Y^†^		PEAK-1Y^†^		PEAK^*^		PEAK + 1Y^†^		PEAK + 2^†^		PEAK + 3^†^	
M	Class I	2.62 (2.63)	*NS*	1.93 (2.06)	*NS*	1.22 (1.65)	*NS*	4.69 (1.71)	0.007	1.6 (2.38)	*NS*	1.31 (1.56)	*NS*	1.42 (2.40)	*NS*
	Class II	2.97		1.08 (1.17)		1.8 (2.12)		5.85 (2.85)		1.62 (1.73)		1.89 (1.76)		0.99 (1.32)	
F	Class I	1.97 (1.95)	*NS*	1.46 (2.08)	*NS*	1.13 (2.07)	*NS*	4.18 (2.35)	NS	0.94 (1.53)	*NS*	1.15 (2.37)	*NS*	1.17 (1.84)	*NS*
	Class II	2.96 (2.47)		1.69 (1.32)		0.96 (1.90)		4.05 (2.33)		0.99 (1.89)		1.79 (1.75)		0.99 (0.62)	

The Mann–Whitney *U*-test shows a significant difference between Class I and Class II only at the peak in females (*P* < .05). Values represent median and interquartile range in brackets.

F, female; M, male; IQR, interquartile range; NS, non-significant.

^*^Significant difference between Peak and Peak+1, Peak-1, Peak+2, Peak-2, Peak+3, Peak-3 (Friedman test, *P* < .05).

^†^Non-significant difference among the 3 years before and 3 years after the peak (Friedman test, *P* > .05).

The significance of the difference between the Co-Gn increments at peak and the preceding and succeeding 3 years was tested for both Class I and Class II samples with the Friedman test with Bonferroni correction. For both samples and for both females and males, the median mandibular increment at the peak is significantly higher than in the three previous and subsequent years (<0.001). The other pairwise comparisons did not show statistically significant results. However, after aligning the data on the highest increment (‘Peak’) rather than the annual increments, a relevant loss of subjects occurred in the Class II sample for the peak −3 years and peak −2 years.

The entity of the peak and the three preceding and following years was compared between Class I and Class II subjects, considering the sexes separately. At the peak, no significant differences were found for Class I and Class II females, while Class II males showed a greater peak (5.85 mm) that Class I males (4.69 mm) (*P* = .007). No other significant differences were found between Class I and Class II patients in the other intervals.

Table 4 shows age at the maximum increment (described as median and interquartile range). No significant differences were found between the sexes in both Class I and Class II sample, and noteworthy variability is expressed by the interquartile range in both categories.

**Table 4.** Descriptive statistics for age at the maximum increment (peak), divided by sex and Class.

		Age at peak	
M	Class I	7 (3)	*NS*
	Class II	6.5 (1)	
F	Class I	7 (3)	*NS*
	Class II	7 (2)	

The Mann–Whitney *U* test found no statistically significant differences between males and females. Values represent median and interquartile range in brackets.

F, female; M, male; NS, non-significant.

## Discussion

The present longitudinal study investigated the presence of a prepubertal mandibular growth spurt in a population of Class I and Class II subjects. Our results suggest that a peak in the 5–11-year interval is consistently present in all subjects for both samples, even though chronological age does not seem to be a suitable indicator for its identification.

When individual mandibular growth peaks were aligned (registered) according to the year of the maximum increment [15, 38], a clear peak was identified of 4.18 mm (2.35) for Class I females and 4.69 (1.71) for Class I males, and of 4.05 (2.33) for Class II females and 5.85 (2.85) for Class II males (Figs 2–3). No significant differences were found between Class I and Class II females, while Class II males showed a significantly greater peak than their Class I counterparts. This result can be interpreted as consistent with evidence that a distal step in the early and mixed dentition does not change its occlusal status in the permanent dentition [42, 43]. As such, it seems that a Class II malocclusion is already present in mixed dentition instead of being established later, thus behaving like a Class I.

For both Class I and Class II patients, and in particular for females, the mean annualized increments seemed to follow a general pattern, with a decreasing intensity of growth until a general minimum and then increasing again. This peak is also accompanied by growth fluctuations occurring before and after the spurt, which can have variable intensity (about 2 mm, ranging from 0.94 mm to 1.97 mm for Class I females and from 1.22 mm to 2.61 mm for Class I males; from 0.96 mm to 2.96 mm for Class II females and from 0.99 mm to 2.97 mm for Class II males).

The median age for the maximum increment was 7 years for Class I and Class II females, 7 years for Class I males and 6.5 years for Class II males. These results are in line with previous investigations that locate the midgrowth spurt at around 7.5 years for both females and males [27, 28], but chronological age has been ruled out multiple times as a reliable indicator for skeletal maturity. In fact, our data show that the age of the maximum increment is quite uniformly distributed through the age intervals. Secular trends must also be taken into consideration when addressing the relationship between chronological age and growth spurts [29, 44]. That being said, the use of records from a collection of historical growth studies (American Association of Orthodontists Foundation’s Craniofacial Growth Legacy Collection; http://www.aaoflegacycollection.org) allowed a longitudinal analysis of yearly records, with the construction of a growth curve for each one of the 115 patients. Furthermore, the inclusion of both Class I and Class II patients offers an unbiased overview and a baseline for future comparative studies.

Among the criticalities of this study is the difficulty, already reported in the literature, to discern between a prepubertal growth spurt and a pubertal one [12]. To classify the peak as a prepubertal or a pubertal one, a reliable growth indicator like those used for the pubertal growth peak is necessary, such as the HWM or the MPM method [14, 15]. In the case of a longitudinal growth study, which would require a prospective enrolment of patients, which is currently not compatible with the ALARA (As Low As Reasonably Achievable) principle. The CVM method [8] received a lot of attention to recognize the onset of the pubertal growth spurt. However, recent studies have shown that the pubertal growth spurt in some individuals can also occur in the prepubertal stages CS1 and CS2, which poses serious problems in the differentiation between a possible prepubertal growth peak and a pubertal one [38]. That said, the exclusion of subjects older than 11 years places us in a relatively ‘safe zone’ for mistaking a pubertal mandibular spurt with an early peak. In fact, a recent cephalometric investigation confirms that the mean age in the record right before the mandibular growth peak was 12.1 years for females and 13 years for males [45].

Another criticality was revealed by the analysis of the method error. The use of the method error for the single Co-Gn measure, which has already been used in the past, can be misleading since it does not reflect the longitudinal nature of data. For this reason, the method errors for the Co-Gn increments were evaluated (Table 1). These values are strongly influenced by the high subjectivity of a cephalometric analysis, which affects single measures, and therefore has a cumulative effect on increments. In this case, this is expressed particularly in the lower bound of the confidence interval, for every age period. Nevertheless, considering mean values, reliability can still be judged as good to excellent for all age intervals (>0.90) except the 5–6 one, which is only moderate (0.85).

The clinical significance of a prepubertal growth peak is still up to question. The unpredictability regarding the age interval at which this peak occurs, and the insufficient predictive value of the CVM method poses some issues regarding the optimal timing of treatment in the individual patient. Despite that, the magnitude of the prepubertal peak detected in this study is high enough that, if correctly detected, it could be valuably used for orthodontic treatment in the future. Clinically speaking, if the prepubertal spurt was univocally identifiable, early therapy could avoid undesired dental compensations and allow the use of two growth peaks instead of just one, thus improving the outcome of treatment. In addition, the onset of the prepubertal growth peak could potentially suggest the onset of the subsequent pubertal growth peak. Future investigations could focus on different growth indicators to establish and predict the timing of this peak.

## Conclusion

Even though the prepubertal growth spurts seem unpredictable in magnitude and timing of occurrence, our data suggest that this peak might be way more common than initially thought. Furthermore, this peak seems to be intense enough to provide the clinician with a good treatment option for both sexes. Future research should focus on the development of methods to improve the recognition of this peak the way it has already been done with the pubertal growth spurt. Valuable help could be obtained from the combination of different growth indicators to increase diagnostic reliability.

## Data Availability

The dataset used and/or analysed during the current study is available from the corresponding author upon reasonable request.
